# CHI3L1 promotes proliferation and improves sensitivity to cetuximab in colon cancer cells by down‐regulating p53

**DOI:** 10.1002/jcla.23026

**Published:** 2019-09-19

**Authors:** Kaitai Liu, Ming Jin, Shuang Ye, Senxiang Yan

**Affiliations:** ^1^ Department of Radiation Oncology The First Affiliated Hospital Zhejiang University of Medicine Hangzhou China; ^2^ Department of Radiation Oncology Lihuili Hospital Ningbo Medical Center Ningbo China; ^3^ Department of Clinical Medicine Ningbo University School of Medicine Ningbo China

**Keywords:** cetuximab, CHI3L1, colon cancer, p53, proliferation

## Abstract

**Background:**

Chitinase 3‐like protein 1 (CHI3L1) is most likely a malignant tumor metastasis‐associated gene. However, the functions of CHI3L1 in colon cancer cell proliferation and its cetuximab sensitivity are still unclear. We aimed to investigate the mechanism of CHI3L1 in promoting colon cancer cell proliferation and its sensitivity to cetuximab.

**Methods:**

The expression of CHI3L1 in colon cancer and adjacent tissues were detected by immunohistochemistry. CHI3L1 was overexpressed in colon cancer cell lines by lentiviral technology. Cell proliferation and sensitivity to cetuximab were measured by MTT assay, cell cycle was analyzed by flow cytometry, and expression of cell cycle‐related proteins was analyzed by immunoblotting.

**Results:**

The results showed that the level of CHI3L1 in colon cancer tissue was significantly higher than that in adjacent tissue, which was also correlated with overall survival. The cell proliferation rate was significantly increased after overexpression of CHI3L1, and the sensitivity to cetuximab was significantly increased. The expression of p53 was down‐regulated while the EGFR was up‐regulated significantly in CHI3L1 overexpressed cells. When rescued the expression of p53 in HCT116‐CHI3L1 cells, the cell proliferation and sensitivity to cetuximab could be restored.

**Conclusion:**

High levels of CHI3L1 are associated with poor prognosis and accelerate the proliferation of colon cancer cells and increase the sensitivity to cetuximab. Its mechanism of increasing the cell proliferation and sensitivity to cetuximab may be explained by down‐regulating p53 expression and then, up‐regulating the expression of EGFR.

## 1 INTRODUCTION

In spite of the decreased incidence of colon cancer (CC) in recent 10 years, it remains the fourth most frequently diagnosed cancer and the second leading cause of cancer death in the United States.^1^ But in China, colon cancer is on the rise, which is the fourth most commonly diagnosed cancers among men and the third one among women.^2^ Approximately 20%‐25% of patients diagnosed with colon cancer present with metastatic disease and result in a poor prognosis.^3^, ^4^, ^5^ Cetuximab is a chimeric monoclonal antibody directed against EGFR that inhibits its downstream signaling pathways. It has been studied in combination with chemotherapy as an initial therapy option for treatment of metastatic colon cancer and provides a clear clinical benefit in patients with RAS wild‐type gene mutation.^6^, ^7^ Unfortunately, cetuximab is only effective in approximately 10%‐20% of patients with colon cancer,^8^, ^9^ implying the sense of identification of biomarkers that predict which patients are likely to be sensitive to cetuximab beyond RAS mutation and BRAF mutation status.

Chitinase 3‐like protein 1 (CHI3L1) (also known as YKL‐40) was discovered in human osteosarcoma cell line MG63 by Johansen et al^10^ in 1992. CHI3L1 is a highly conserved glycoprotein with a coding gene of about 8 kb located on human chromosome 1q32.1.^11^, ^12^ CHI3L1 is highly expressed in embryonic tissues with rapid proliferation and differentiation characteristics and adult tissue cells with high cell activity.^13^, ^14^ Several studies have demonstrated that plasma YKL‐40 is a potential prognostic marker in different solid tumors, such as breast cancer, ovarian cancer, prostate cancer, and colon cancer.^15^, ^16^, ^17^, ^18^, ^19^, ^20^, ^21^ Besides, CHI3L1 has been shown to promote cancer angiogenesis in breast, colon, and glioblastoma cancer cells in vivo.^22^, ^23^, ^24^ These results suggest that CHI3L1 is most likely a malignant tumor metastasis‐associated gene. However, the functions of CHI3L1 in colon cell proliferation and its sensitivity to cetuximab are still unclear.

In the present study, we aimed to investigate the mechanism of CHI3L1 in promoting proliferation and sensitivity to cetuximab in colon cancer cells.

## 2 MATERIALS AND METHODS

### 2.1 Reagents and cell lines

HCT116, SW620, and 293T cells were provided by Dr Ye Xiaolei from Ningbo Medical Science Research Institute. MTT (AMRESCO); DMEM medium (Thermo Fisher); Fetal bovine serum (FBS, GIBCO); DMSO (Life science); Cetuximab (Merck & Co, Inc); Streptomycin (GIBCO); X‐Treme transfection reagent; Annexin V‐APC /7‐AAD Dye Kit; 7500 Fast fluorescence quantitative PCR instrument; Gel imaging system (Shanghai Tianneng, Tanon 2500); Antibodies including anti‐EGFR, anti‐p53, anti‐β‐actin, and HRP‐labeled secondary antibody were all purchased from Cell Signaling Technology; CHI3L1 reconstruction protein (Novoprotein); CMax Plus microplate reader (Molecular Devices), Attune flow cytometer (Applied biosystems); Electrophoresis and blotting system (Bio‐Rad).

### 2.2 Clinical data

The relative expression levels of CHI3L1, as well as clinicopathological and survival data for 285 cases of colon cancer patients and 40 adjacent healthy tissues, were downloaded from the UCSC Xena platform for public and private cancer genomics data visualization and interpretation (https://xena.ucsc.edu/). The Ningbo Medical Center Lihuili Hospital Ethics Review Board approved the study. The clinicopathological variables of 243 colon cancer cases for CHI3L1 expression were summarized in Table 1.

**Table 1:** The correlation of Chitinase 3‐like protein 1 (CHI3L1) expression with clinicopathologic characteristics of CC patients

Variables	Cases (n)	CHI3L1		*P* value
		High	Low	
Total	243	218	25	
Age (y)				
≥60	164	151	13	
<60	79	67	12	.113
Gender				
Male	133	121	12	
Female	110	97	13	.528
Pathological stage				
I/II	138	124	14	
III/IV	105	94	11	1.000
T stage				
T1/T2	48	43	5	
T3/T4	195	175	20	1.000
N stage				
Negative	142	128	14	
Positive	101	90	11	.832
M stage				
Negative	170	154	16	
Positive	73	64	9	.496

### 2.3 Immunohistochemistry (IHC) analysis

The tissue microarray of colon cancer was purchased from the Servicebio Biotech Co, LTD (IWLT‐N‐82C74), and it included 15 colon cancer samples and corresponding 15 adjacent normal tissues. Anti‐CHI3L1 antibody (ab156222, Rabbit polyclonal antibody, Abcam) was used for IHC detection of the expression of CHI3L1 protein in tissue microarray according to the process as follows: IHC examinations were carried out on 3 mm thick sections. For anti‐LATS1 IHC, unmasking was performed with 10 mmol/L sodium citrate buffer, pH 6.0, at 90°C for 30 minutes. Sections were incubated in 0.03% hydrogen peroxide for 10 minutes at room temperature, to remove endogenous peroxidase activity, and then in blocking serum (0.04% bovine serum albumin, A2153, Sigma‐Aldrich and 0.5% normal goat serum X0907, Dako Corporation, in PBS) for 30 minutes at room temperature. The anti‐CHI3L1 antibody was used at a dilution of 1:200 and was incubated with slides overnight at 4°C. Sections were then washed three times for 5 minutes in PBS. Non‐specific staining was blocked with 0.5% casein and 5% normal serum for 30 minutes at room temperature. Finally, staining was developed using diaminobenzidine substrate, and sections were counterstained with hematoxylin. Normal serum or PBS was used to replace the CHI3L1 antibody in the negative control.

The proportion score and an intensity score were semi‐quantitatively counted, respectively. The proportion score according the percentage of positive cells (0, none; 1, ≤10%; 2, 11%‐25%; 3, 26%‐50%; 4, >50%), and the intensity score based on the coloring strength (0, no staining; 1, weak; 2, intermediate; 3, strong). The expression level of CHI3L1 was as the total immunostaining scores, which were calculated as the product of proportion and an intensity score. Finally, a total score was obtained, ranging from 0 to 12. Based on the analysis in advance, CHI3L1 expression was categorized into negative (score 0), weak (score 1‐3), intermediate (score 4‐6), and strong (score 7‐12). The scoring was independently assessed by two pathologists.

### 2.4 Cell culture

Monolayer cells were cultured in DMEM medium containing 10% FBS, 100 U/mL penicillin, and 100 U/mL streptomycin at 37°C with 5% CO_2_, and the cells were sub‐cultured every 2‐3 days at a ratio of 1:3‐1:5. Cell growth at the logarithmic phase was used in experiments.

### 2.5 CHI3L1 overexpression plasmid construction

A full‐length cDNA primer was designed based on the human CHI3L1 gene, and an enzyme cleavage site was added. The sequence of the primer was as follows: 5′‐GCT CTA GAA TGG GTG TGA AGG CGT CTC A‐3′, 5′‐CGG GAT CCC TAC GTT GCA GCG AGT GCA T‐3′. The complete gene was amplified by RT‐PCR, and the PCR product was digested with Xba I and BamH I endonucleases and ligated into the pCDH‐CMV‐GFP‐puro lentivirus plasmid by T_4_ ligase. About 5 μL of the ligation plasmid was added to Stbl3 competent cells. The mixture was placed on the ice for 30 minutes, followed by heat‐shocking for 90 seconds at 42°C and then placed back on the ice rapidly for 2 minutes. Each tube was incubated with LB medium (Amp^−^) for 1 hour at 37°C, and the moderate bacterial solution was smeared to LB plate (Amp^+^) and cultured at 37°C for 12‐16 hours. Isolated colonies were identified by PCR and double enzyme digestion. Positive colonies were sequenced, and the correct recombinant plasmid pCDH‐CMV‐CHI3L1‐GFP‐puro was selected.

### 2.6 Lentivirus packaging and infection

293T cells were cultured in DMEM containing 10% fetal calf serum (FBS), 100 U/mL ampicillin, and 100 U/mL streptomycin based on a 37°C 5% CO_2_ incubator. The culture solution was changed to DMEM medium 2 hours before the transfection. About 2‐3 μg of recombinant lentiviral plasmid pCDH‐CMV‐CHI3L1‐GFP‐puro (pCDH‐CMV‐GFP‐puro plasmid used as control), lentiviral packaging plasmid △8.2, and VSV‐G were mixed at a weight ratio of 10:10:1. About 6 μL of transfection reagent and 100 μL of Opti‐MEM medium (Serum‐free and antibiotic‐free) was added into the mixture, which was then incubated at room temperature for 20 minutes. The mixture was added to the cultured 293T cells, and after transfection for 6 hours, the medium was replaced with complete medium. After 48 hours of transfection, the fluorescence was observed, and the supernatant virus was collected and pass through a 0.45 μm filter to SW620 and HCT116 cells. The cells were screened with puromycin to obtain CHI3L1 stably expressed cell line. Quantitative PCR (qPCR) and Western blotting were used to detect the expression of CHI3L1 in overexpressed and control cells.

### 2.7 Real‐time PCR

To detect CHI3L1 mRNA expression in different cell lines. Cell total RNA was extracted with Trizol (method according to the instruction) and was reverse transcribed to cDNA by M‐MLV reverse transcriptase. CHI3L1 primers were as follows: CHI3L1‐F:5′‐GAT GTG ACG CTC TAC GGC AT‐3′, CHI3L1‐R:5′‐TGA TAA AGT CCG GCG ACT C‐3′; GAPDH‐F:5′‐GAG AAG GCT GGG GCT CAT TTG‐3′, GAPDH‐R: 5′‐GGT GCT AAG CAG TTG GTG GT‐3′. The reaction system was as follows: template cDNA 500ng, both upstream and downstream primers 0.4 μL, 50 × ROX II 0.4 μL, 2 × SYBR green master mix 10 μL, and deionized water was supplemented to 20 μL. PCR reaction conditions were as follows: heated start at 95°C for 2 minutes, degeneration at 94°C for 30 seconds, annealing at 60°C for 30 seconds, extension at 72°C for 30 seconds, reaction lasted for 40 cycles. The melting curve was based on the instrument default procedure. Each sample was set triplicate wells. The relative expression level of CHI3L1 was calculated by the 2^−ΔΔ^ *^C^* ^t^ method.

### 2.8 Western blot

Removed the cells from CO_2_ incubator, discarded the medium and put the cells on the ice, rinsed cells by cold PBS for two times, drained the remaining PBS, and lysed cell on the ice in the RIPA lysate. The cell lysate was passed through a 1 mL syringe to make DNA fracture, and 3 μL of cell lysate was taken for BCA quantification after incubated at 95°C for 5‐10 minutes. About 40 μg of protein with the sample buffer was loaded in each lane for electrophoresis in 10% SDS‐page gel. Protein was then electric transferred to PVDF membrane by 200 mA constant‐current for 1.5 hours. PVDF membrane was blocked using 5% skim milk for 1 hour and incubated with primary antibody at 4°C overnight. The membrane was washed with TBST for three times, added with the secondary antibody and incubated at room temperature for 1 hour Then, the membrane was washed with TBST for three times. The membrane was exposed to enhanced chemiluminescence substrates, and blots were photographed. The relative expression of proteins was quantified by ImageJ software.

### 2.9 MTT assay

Using MTT assay to detect cell sensitivity to cetuximab. About 24 hours before drug treatment, HCT116 and SW620 cells were trypsinized and collected, and the cell density was adjusted to 5 × 10^4^ cells/mL. About 100 µL of cells were inoculated in 96‐well culture plates per well. Cells were treated with different concentrations of cetuximab: 0, 0.25, 0.5, 1, and 2 mg/mL, each concentration with five repeats. After 48 hours, 20 μL of MTT (5 mg/mL) was added to each well and incubated for 4 hours at 37°C. Then, 150 μL of DMSO was added to each well after the supernatant was discarded. The absorbance at 562 nm was measured with 630 nm as a reference wavelength.

### 2.10 Cell cycles analysis

When the cells were grown to a confluence of 80%, the cell culture supernatant was discarded. The cells were washed once with PBS, and the cells were digested with trypsin. The collected cells were centrifuged at 1000 *g* for 5 minutes, and the supernatant was discarded. The cells were washed once with ice‐cooled PBS, centrifuged at 1200 *g* for 5 minutes, fixed in ice‐cooled 70% ethanol at 4°C for 1 hour, centrifuged at 1200 *g* for 5 minutes to remove ethanol, washed the cell pellet once with PBS, and resuspended in 500 μL of PI staining solution. The cell suspension was filtered by 300 mesh screen and detected on a flow cytometer.

### 2.11 Statistical analysis

The intergroup comparison of clinicopathologic variables was performed with the chi‐square test. Overall survival (OS) was calculated from the date of diagnosis to the date of death for any cause. Survival was analyzed using the Kaplan‐Meier method. The association between each of the potential prognostic factors and differences between the curves were analyzed by the log‐rank test. Multivariate analysis was performed using the Cox regression model. The statistical test was two‐sided, and *P < *.05 was considered statistically significant. Statistical analyses were conducted by SPSS 20.0 (IBM, SPSS) and GraphPad Prism.

## 3 RESULTS

### 3.1 CHI3L1 levels are up‐regulated in colon cancer and correlated with unfavorable prognosis of colon cancer patients

**Figure 1:**
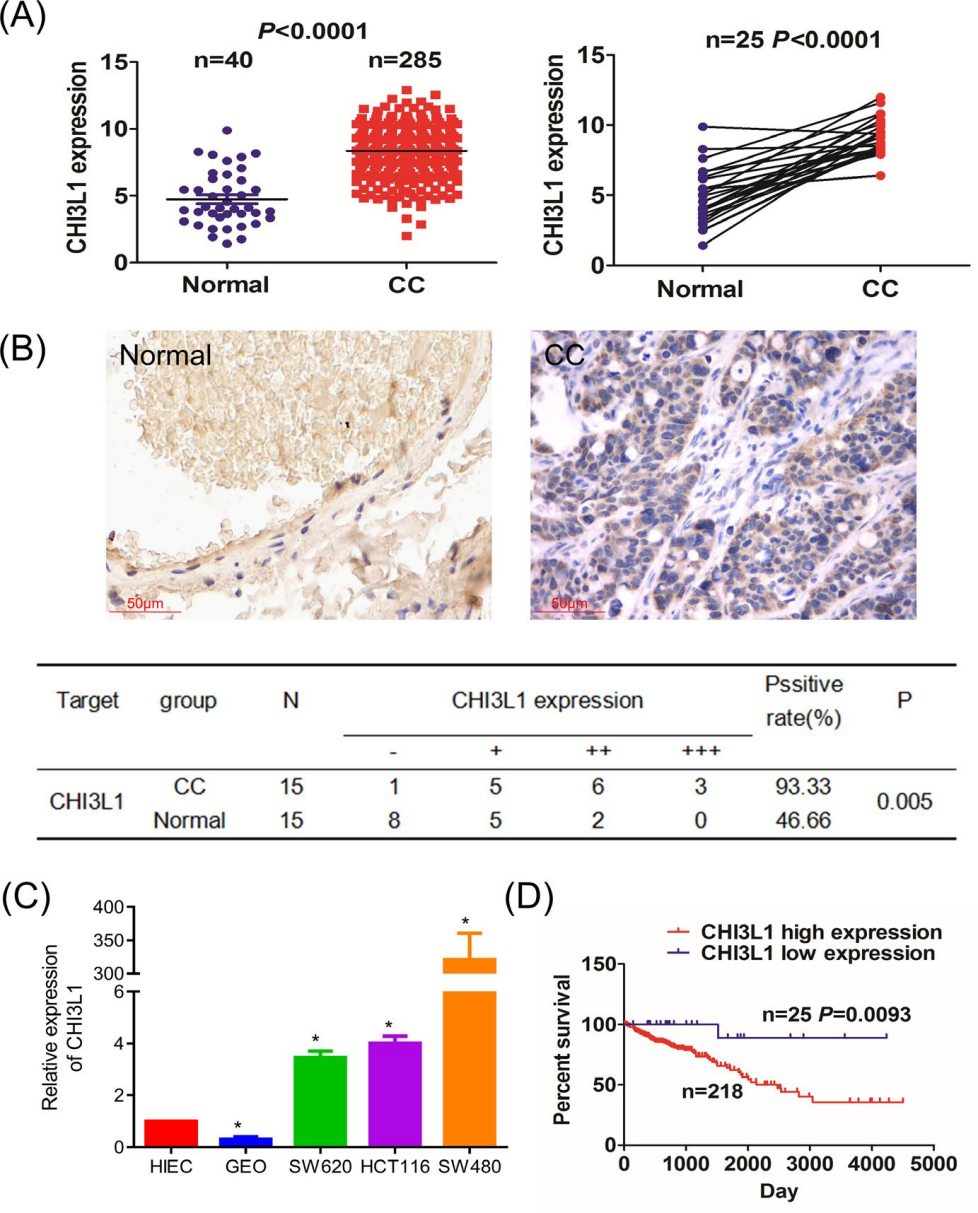
Chitinase 3‐like protein 1 (CHI3L1) expression was associated with overall survival in patients with CC. A, The UCSC Xena platform cohort analysis of the differentially expressed levels of CHI3L1 in the total CC samples as well as in the pair‐matched tumor tissues. B, IHC analysis of the expression level and cellular localization of CHI3L1 in CC tissue cells. C, qPCR analysis of the mRNA expression level of CHI3L1 in CC cell lines. D, Kaplan‐Meier analysis of the association of CHI3L1 high or low expression with the overall survival of the patients with CC.*, *P* < .05

By using the UCSC Xena platform, we demonstrated that the expression level of CHI3L1 was significantly increased in colon cancer tissues (n = 285, *P* < .0001). In the 25 pair‐matched samples, CHI3L1 was markedly up‐regulated in cancer tissues compared with adjacent normal tissues (n = 25, *P* < .0001; Figure 1A). The CHI3L1 immunohistochemistry staining in 15 colon cancer samples and corresponding adjacent normal tissues showed that CHI3L1 mainly distributed in the cytoplasm and its positive rate was much higher in cancer tissues than adjacent normal tissues (*P* = .005; Figure 1B). Besides, CHI3L1 mRNA expression was also higher in three colon cell lines than the normal intestinal epithelium cell line HIEC except for the colon cell line GEO by using qRT‐PCR analysis (Figure 1C). These results indicated that the upregulation of CHI3L1 was a frequent molecular event in colon cancer.

Furthermore, we analyzed the correlation of the clinicopathological characteristics with the CHI3L1 expression according to the 243 colon cancer cases downloaded from the UCSC Xena platform. The result showed that the expression of CHI3L1 had no significant correlation with factors such as age, gender, pathological stage, and TNM stage (all *P* > .05, Table 1). Patients with high CHI3L1 expression had significantly better overall survival (OS) time than those with low CHI3L1 expression by Kaplan‐Meier analysis (Figure 1D). Moreover, III/IV pathological stage, positive N stage, positive M stage, and high CHI3L1 expression were identified as significant risk factors for poor survival on univariate analysis (all *P < *.05). When multivariate analysis with Cox regression was performed, we convinced M stage and CHI3L1 expression as independent prognostic factors (all *P < *.05; Table 2).

**Table 2:** Cox regression analysis of Chitinase 3‐like protein 1 (CHI3L1) expression as survival predictor

Variables	Univariate Cox regression analysis		Multivariate Cox regression analysis	
	RR (95% CI)	*P* value	RR (95% CI)	*P* value
Age (y)				
<60 vs ≥60	1.473 (0.807‐2.688)	.207	NA^a^	NA^a^
Gender				
Male vs Female	1.477 (0.873‐2.498)	.146	NA^a^	NA^a^
Pathological stage				
III/IV vs I /II	2.609 (1.542‐4.416)	<.0001	2.048 (0.439‐9.552)	.361
T stage				
T3 + T4 vs T1 + T2	1.751 (0.749‐4.095)	.196	NA^a^	NA^a^
N staging				
Positive vs Negative	2.485 (1.475‐4.186)	.001	1.139 (0.253‐5.135)	.866
M stage				
Positive vs Negative	2.435 (1.444‐4.108)	.001	1.907 (1.106‐3.289)	.020
CHI3L1 expression				
High vs Low	8.780 (1.215‐63.461)	.031	9.317 (1.288‐67.393)	.027

a Not analyzed.

### 3.2 Construction of CHI3L1 stably transfected cell line

**Figure 2:**
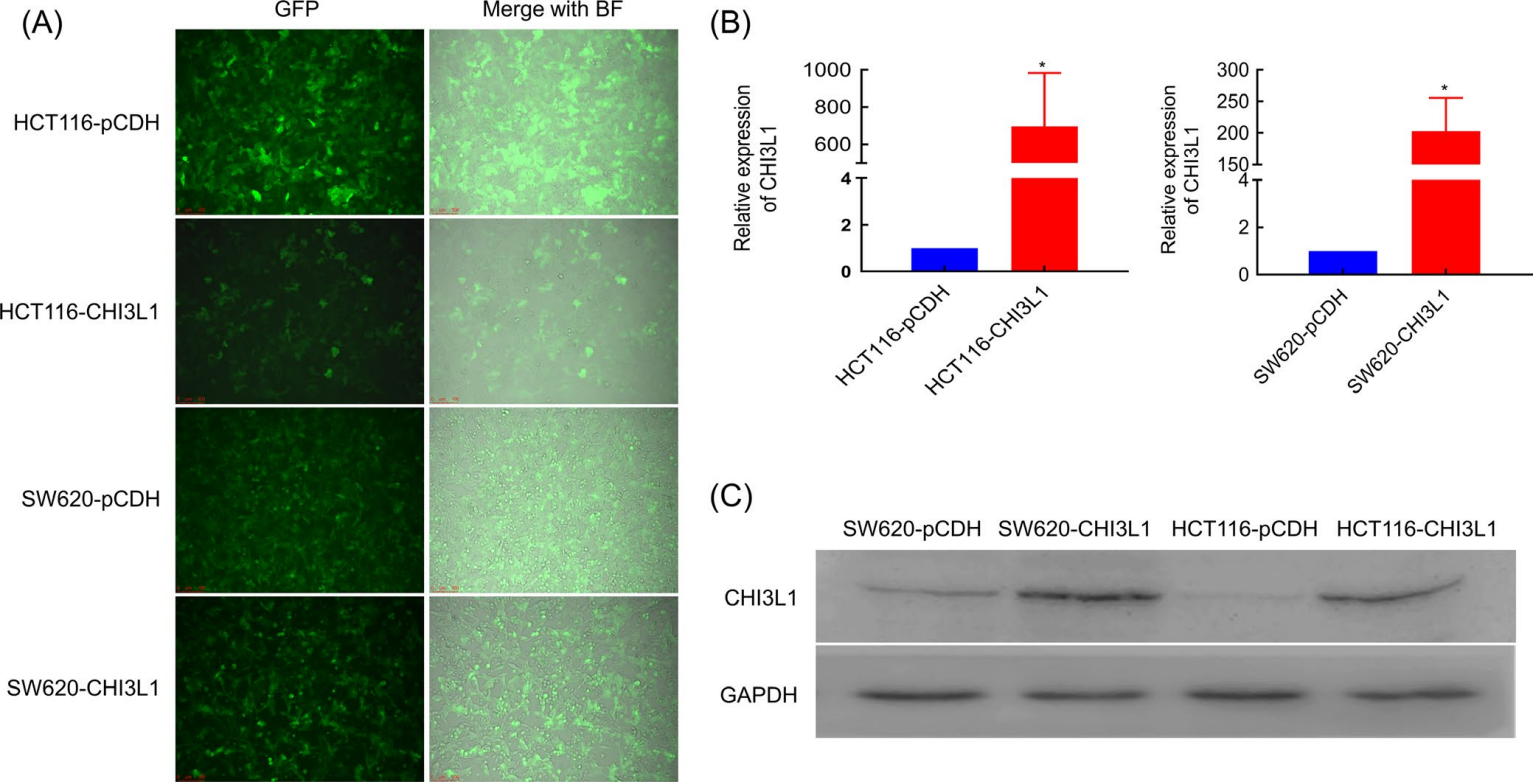
The infection efficiency of Chitinase 3‐like protein 1 (CHI3L1) in SW620 and HCT116 cells. A, The fluorescent intensity of CC cells after infection for 48 h; Left: green fluorescence field; Right: bright field merged with green fluorescence field. B, CHI3L1 mRNA expression and C, Protein expression in different groups. Three replicates were set for each group in qPCR assay. *, *P* < .05

The CHI3L1 stably transfected cell line was constructed by lentiviral technology. The pCDH‐CHI3L1 overexpression plasmid was successfully constructed by sequencing. CHI3L1 was overexpressed in SW620 and HCT116 by lentiviral technology, and the cell infection rate was 98% from the fluorescence results (Figure 2A). qPCR results showed that the level of CHI3L1 mRNA was significantly increased in SW620 and HCT116 cells after transfection of the expression plasmid (Figure 2B). The results of immunoblotting showed that the expression of CHI3L1 was significantly increased compared with the control (Figure 2C), which indicates that CHI3L1 Stable transfected cell lines were successfully constructed.

### 3.3 Increased sensitivity to cetuximab by overexpression of CHI3L1

**Figure 3:**
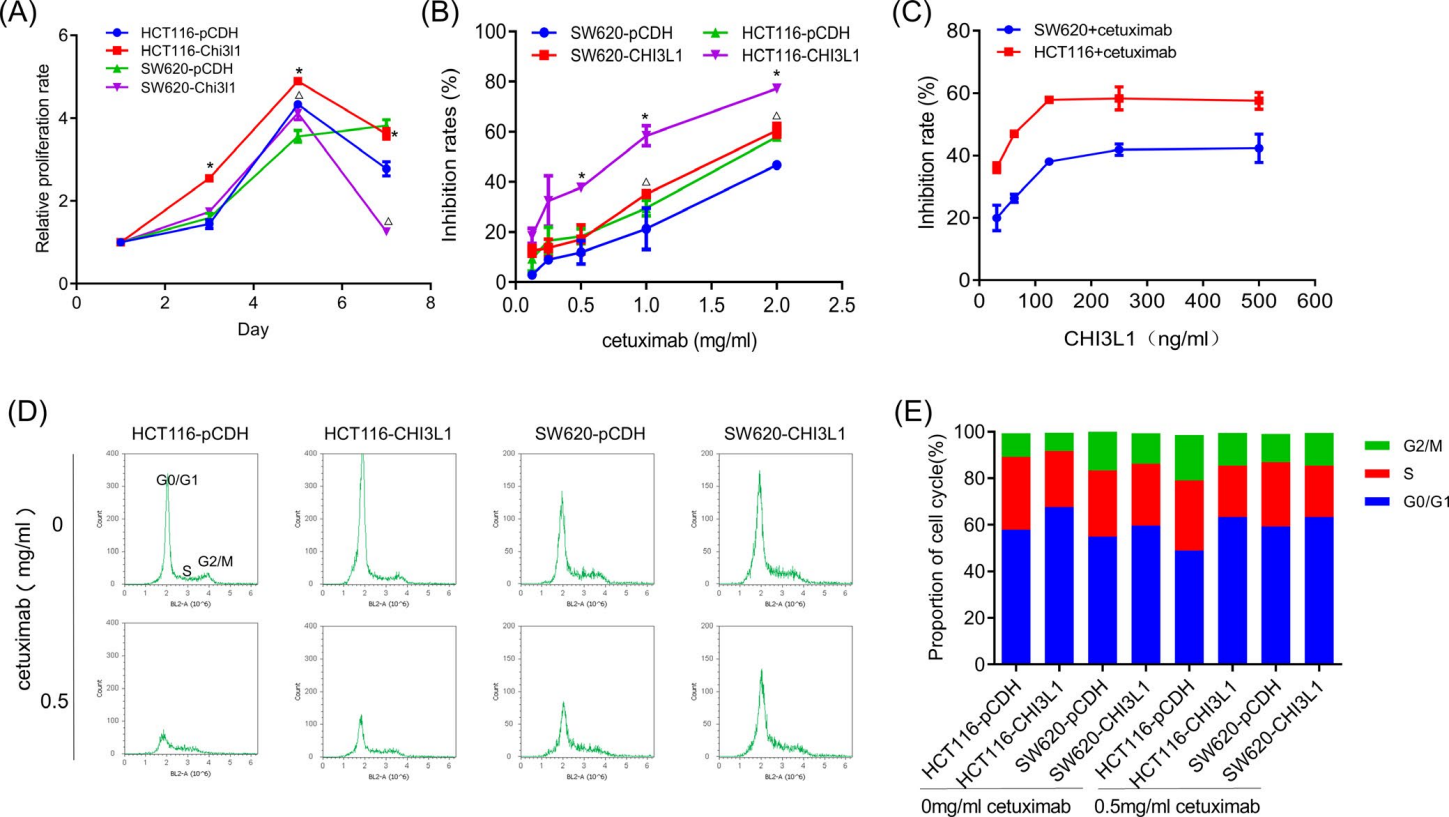
Effect of Chitinase 3‐like protein 1 (CHI3L1) overexpression on cell proliferation and drug sensitivity. A, CHI3L1 overexpression promotes cell proliferation. B, The inhibition rates of SW620 and HCT116 cells after treatment with different concentrations of cetuximab. C, The sensitivity of cells to cetuximab (1mg/ml) increased after the addition of different concentrations of exogenous CHI3L1. D, Effect of cetuximab on cell cycle after overexpression of CHI3L1. E, Proportion of each phase of the cell cycle. *, *P* < .05, in HCT116 group; ^△^, *P* < .05, in SW620 group

The cell proliferation curve was examined, and the results showed that the relative proliferation rate of cells increased after overexpression of CHI3L1 (Figure 3A). The sensitivity of the cells to cetuximab was examined. The results showed that the inhibition of cetuximab by HCT116 overexpressed CHI3L1 was significantly enhanced (Figure 3B). About 31.25, 62.5, 125, 250, and 500 ng/mL of exogenous CHI3L1 protein were added to the two colon cancer cells, and the sensitivity of which to cetuximab was detected. The results showed that the sensitivity of cells to cetuximab increased with the increase of CHI3L1 in the concentration range of 31.25‐250 ng/mL, indicating that high level of CHI3L1 will increase the sensitivity of colon cancer cells to cetuximab (Figure 3C). The effect of cetuximab on the cell cycle of colon cancer cells before and after CHI3L1 overexpression was detected by flow cytometry. Compared with the control group, the proportion of G0/G1 phase cells was increased in the CHI3L1 overexpressed group both in the presence and absence of cetuximab treatment (Figure 3D and 3).

### 3.4 Mechanism exploration

**Figure 4:**
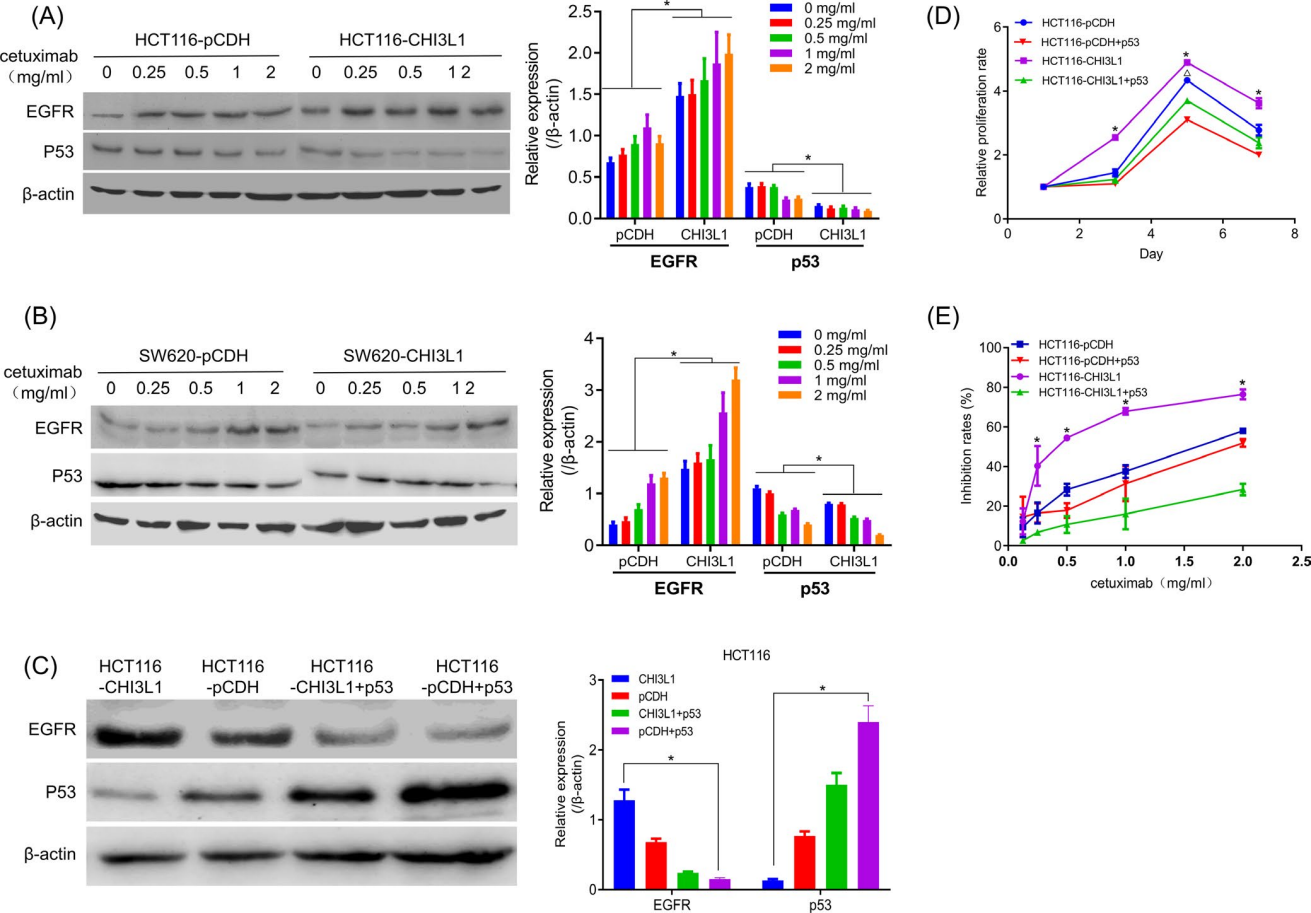
Mechanism of Chitinase 3‐like protein 1 (CHI3L1) overexpression affecting cell sensitivity to cetuximab. A, The expression of EGFR and p53 in HCT116‐pCDH and HCT116‐CHI3L1 cells after treatment with cetuximab. B, The expression of EGFR and p53 in SW620‐pCDH and SW620‐CHI3L1 cells after treatment with cetuximab. C, The expression level of EGFR and p53 after transfection of p53 overexpression plasmid in HCT116‐CHI3L1 cells. D, p53 overexpression inhibited cell proliferation. E, Overexpression of p53 decreased the sensitivity of HCT116‐pCDH cell to cetuximab. *, *P* < .05, in CHI3L1 group; ^△^, *P* < .05, in pCDH group

The changes in cell cycle‐related proteins were detected by immunoblotting. The results showed that with the increase of cetuximab, the expression of EGFR up‐regulated while the expression of p53 protein was significantly down‐regulated (Figure 4A and 4). It is speculated that CHI3L1 may increase the sensitivity of cells to cetuximab by down‐regulating p53 and up‐regulating EGFR. To confirm this hypothesis, p53 overexpression plasmid was transfected into HCT116‐CHI3L1 cells to increase the expression level of p53. Its proliferation and sensitivity to cetuximab were detected, and the expression levels of p53 and EGFR were also detected by Western blot. The results showed that p53 was down‐regulated and EGFR was up‐regulated after overexpression of CHI3L1, while the expression of EGFR was decreased after the overexpression of p53 (Figure 4C). At the same time, the relative proliferation rate (Figure 4D) and the sensitivity of cells to cetuximab were significantly decreased (Figure 4E) after the overexpression of p53 compared with the control group. These indicated that CHI3L1 could increase the cell sensitivity to cetuximab and promote the proliferation of cell by down‐regulating p53 and up‐regulating EGFR.

## 4 DISCUSSION

Colon cancer is one of the common malignant tumors, which seriously threatens human life and health. Chemotherapy, combined with targeted therapy, is currently the main treatment for colon cancer. Cetuximab is one of the most commonly used targeted drugs for the treatment of colon cancer. It is often combined with other drugs as the main therapeutic drug for metastatic colon cancer.^25^, ^26^ CHI3L1 is a new marker found in cancer patients. High levels of CHI3L1 in plasma in humans are associated with gastrointestinal cancer and risk of death.^27^, ^28^ Tarpgaard et al^20^ reported that the survival rate of colon cancer patients with high plasma levels of CHI3L1 was significantly lower than those of low plasma CHI3L1, indicating that CHI3L1 was a poor prognosis biomarker. It is worth noting that although the result showed that plasma CHI3L1 concentration was an independent prognostic factor for the metastatic colon cancer patients, subgroup analysis showed that in patients combined with cetuximab, CHI3L1 concentration was not significantly related to progression‐free survival (PFS), indicating that patients with high expression of CHI3L1 might benefit from cetuximab. In this study, we investigated the mechanism of CHI3L1 in promoting colon cancer cell proliferation and its correlation with the sensitivity of cetuximab.

Lentiviral technology is a useful tool for studying gene function; that is, the gene is overexpressed in cells by an artificial lentivirus‐engineered skeleton. It has been reported that silencing of CHI3L1 using siRNA in cells has shown that a decrease in CHI3L1 levels in U87MG cells can significantly reduce cell viability.^29^ In this study, two colon cancer cells were successfully infected with lentivirus, which could overexpress CHI3L1 stably, and laid a foundation for downstream biological function research.

Chitinase 3‐like protein 1 is mainly produced by cancer cells, macrophages, and neutrophils and is highly expressed in various malignant tumors. In this study, qPCR was used to detect the expression level of CHI3L1 in colon cancer cells, and the highest expression level was found in HCT116 and SW620 cells, which provided a basis for subsequent rectal cancer cell screening. Ngernyuang et al^30^ revealed that CHI3L1 plays a crucial role in Vasculogenic mimicry, which may contribute to tumor invasion of cervical cancer. Chen's analysis showed that tumor recruitment of M2 macrophages promoted gastric cancer and breast cancer metastasis by secreting CHI3L1 protein.^31^ In this study, CHI3L1 was successfully overexpressed in SW620 and HCT116 cells. The results of the cell proliferation curve showed that the relative proliferation rate of cells increased significantly after overexpression, which indicated that the high expression of CHI3L1 could promote the proliferation of colon cancer cells.

Cetuximab is currently a commonly used targeted drug for the treatment of metastatic colorectal cancer. In this study, CHI3L1 was overexpressed in colon cancer cells by lentiviral technology, and the sensitivity of colon cancer cells to cetuximab was evaluated. The results showed that the sensitivity to cetuximab was significantly improved both in CHI3L1 overexpressed and exogenous CHI3L1 added colon cancer cells, indicating that high levels of CHI3L1 could increase the sensitivity of cells to cetuximab, which was consistent with the results of Tarpgaard's study.

The p53 gene is one of the most frequently studied tumor suppressor genes, which is associated with malignant transformation as well as tumorigenesis and development. There is a high incidence of mutations or deletions of this gene in most tumor types.^32^ Also, studies indicated that downregulation of p53 could accelerate the proliferation in cancer cells with various mechanisms.^33^, ^34^, ^35^ In our study, overexpression of CHI3L1 in colon cancer cells stimulated cell proliferation, which could be partly explained by down‐regulation of p53. EGFR was a key target for molecular therapy, involved in the regulation of cell metabolism, growth, migration, and differentiation, which was overexpressed in various tumors such as prostate cancer, breast cancer, and colon cancer.^36^ Meanwhile, a previous study showed that loss of p53 could cause the increased activity of EGFR promoter, and further increase the expression of EGFR.^37^ In consistence with the result, our study showed that EGFR was up‐regulated accompanied by down‐regulation of p53 in CHI3L1 overexpressed cells. Further, when the p53 overexpression plasmid was transferred to rescue the level of p53, cell proliferation and sensitivity to cetuximab were significantly restored. This study revealed that high levels of CHI3L1 could increase the sensitivity of cetuximab by down‐regulating p53 and up‐regulating EGFR, which might provide valuable drug guidance for cetuximab in patients with colon cancer.

In conclusion, high levels of CHI3L1 are associated with poor prognosis in colon cancer patients. Overexpression of CHI3L1 can promote the proliferation of colon cancer cells and increase the sensitivity to cetuximab by down‐regulating the p53 expression and up‐regulating the expression of EGFR. In the future, we will further verify the role of CHI3L1 in guiding the personalized medicine of cetuximab.
